# Study on Applicability of Energy-Saving Conductors in Alpine Regions †

**DOI:** 10.3390/ma19050828

**Published:** 2026-02-24

**Authors:** Wenqi E, Haodong Liu, Cong Zeng

**Affiliations:** 1 School of Civil Engineering and Architecture, Northeast Electric Power University, Jilin 132012, China; 2 Huizhou Boluo Power Supply Bureau of Guangdong Power Grid Co., Ltd., Huizhou 516100, China

**Keywords:** energy-saving conductors, cold environment, finite element model, transmission efficiency

## Abstract

The development of energy-efficient conductors capable of operating reliably in harsh, cold climates is crucial for sustainable power infrastructure. High-mountain and cold regions are key research scenarios for energy-saving conductors, enabling the natural enhancement of conductor heat dissipation in low-temperature environments and improving the current carrying capacity and energy efficiency. These regions are rich in renewable energy and urgently need efficient transmission channels. However, the extremely complex working conditions create strict requirements for the thermal–mechanical coupling performance of conductors, and existing research has paid insufficient attention to this. This study evaluates the thermal and mechanical performance of three advanced energy-saving conductors (JLHA3-275, JL1/G1A-240/30, JL/LHA1-135/140) in comparison with a conventional conductor (JL/G1A-240/30) under cold-region operating conditions. A finite element analysis model, validated against theoretical calculations under combined meteorological factors, was employed to simulate radial temperature fields and stress distribution. The results demonstrate that the JLHA3 conductor exhibits superior heat dissipation and minimal resistive losses, maintaining a radial temperature of −23.35 °C under a 700 A load, approximately 1.6 °C lower than the conventional type. Its temperature further decreases significantly with increased wind speeds. Thermally, JLHA3 shows high stability across a broad temperature range (−28.85 °C to 29.03 °C). Mechanically, it displays uniform stress distribution and a notable decrease in stress from 79.53 MPa to 39.46 MPa with rising temperatures, indicating excellent flexibility and thermal adaptability. These findings confirm that the JLHA3 conductor offers an optimal combination of thermal performance, structural reliability, and energy efficiency for high-altitude, cold-region power transmission applications.

## 1. Introduction

To meet the demands of green and low-carbon transformation in the power industry, energy-efficient conductors are gradually replacing conventional ones in high-voltage transmission lines [1]. Currently, their application is predominantly concentrated in Southern China, while research on the operational characteristics of such conductors under the cold climatic conditions of northern regions remains insufficient. Environmental factors significantly influence the temperature variation of overhead lines [2], and temperature fluctuations are closely related to stress changes in these lines [3]. Therefore, accurately analyzing the stress variation patterns of conductors under low temperature and high-altitude conditions, and conducting comprehensive coupled simulations of stress and temperature, are of great significance for enhancing the economy, safety, and reliability of power grid construction. The synergistic analysis of stress and temperature fields can effectively improve the transmission capacity, reduce line losses, and ensure the efficient operation of transmission lines under various environmental conditions.

The research and deployment of energy-saving conductors in high-altitude and cold regions have outstanding strategic significance but face scientific challenges. Firstly, strengthening the heat dissipation of conductors in low-temperature environments can fully leverage the low resistance advantage of energy-saving conductors, achieving a lower temperature rise or higher current carrying capacity, which is difficult to replicate in mild regions. Secondly, a large number of clean energy bases around the world are concentrated in high-altitude cold regions, and high-performance conductors are the core guarantee for building efficient and reliable transmission channels and supporting the “dual carbon” goal. Thirdly, extreme environmental stress in mountainous areas poses a thermal–mechanical coupling challenge for conductor materials, and existing current carrying capacity calculation standards are difficult to adapt to new composite energy-saving conductors, which can easily lead to performance misjudgment. Therefore, conducting multi-physics coupling simulations and performance evaluations in extreme environments is not only urgently needed in engineering but would also fill the gap in research on extreme climate adaptability.

Currently, methods for analyzing the operating temperatures and current carrying capacities of overhead transmission lines are primarily based on numerical calculations of thermal balance equations and finite element analyses, such as those adopted in ICE1597-1995, the Morgan method, and the IEEE-738 standard [4]. Complementing these, the CIGRE Technical Brochure 601 (TB-601) offers a detailed methodology for thermal rating, encompassing both steady-state and dynamic scenarios, and is a cornerstone document for many international utilities. While CIGRE TB-601 enables the more advanced treatment of some heat transfer phenomena, like the aforementioned standards, it fundamentally relies on a homogenized thermal model of the conductor. Chawasak et al. [5] further incorporated factors such as the ambient temperature, wind speed, wind direction angle, and solar radiation to improve the accuracy of conductor temperature estimation. Yang et al. [6] established a radial temperature gradient model for conductors, revealing the influence of internal air gaps on the temperature distribution, and employed electrothermal coupled numerical calculations to analyze the effects of the wind speed, ambient temperature, and current on the temperature rise. Zhao et al. [7] developed a mathematical model for the temperature fields of overhead conductors, simulating the heat dissipation of different conductor types under natural and forced convection conditions. Their study derived the relationship between the conductor temperature, current carrying capacity, and wind speed, indicating an approximately linear correlation between the conductor temperature and ambient temperature. Guo et al. [8] analyzed the flow fields around conductors and their impacts on the temperature field using a fluid–solid coupling model. Beňa Ľ et al. [9] proposed a temperature calculation method for overhead conductors that accounts for the corona effect and compared the temperature rise patterns under different weather conditions, considering the ambient temperature, solar irradiance, wind speed, and wind direction. However, existing studies often treat conductors as homogeneous entities, failing to fully consider the non-uniform thermal distribution caused by their layered structure, which leads to certain errors in the calculation of the conductor temperature and current carrying capacity.

The standardized conductor temperature estimation methods derived from the thermal equilibrium equation have their own assumptions and applicable ranges, and understanding their characteristics is crucial for research. IEEE 738-2012 [4] is the most widely used steady-state model; it combines Joule heating, convective/radiative cooling, and solar heating. Empirical formulas have been verified through actual measurements, but the homogenization assumption ignores the radial temperature gradient of the stranded conductor, which can easily lead to the misjudgment of the peak temperature and current carrying capacity. The physical basis of the Morgan method in the UK is similar, but the empirical coefficients of convective radiation are different, resulting in more conservative calculation results. Similarly, due to the simplification of the conductor structure, it is not suitable for the analysis of complex composite energy-saving conductors. IEC 61597:1995 [4] is a general reference framework that defines the rated current under standard conditions. Non-reference operating parameters need to be corrected in conjunction with other standards, and there is a lack of dynamic environmental guidance, making it unsuitable for real-time dynamic current carrying assessment.

In summary, while IEEE 738, the Morgan method, and IEC 61597 all stem from the same energy balance principles, they differ significantly in their empirical correlations, levels of detail, and regional adoption. Critically, all three conventional models share a common and significant limitation: they model the conductor as a thermally homogeneous entity. This assumption fails to capture the true physics of heat generation and dissipation within the multi-layered, multi-material structures of modern conductors, where internal air gaps, contact resistances, and differing thermal conductivities between steel, aluminum, and alloy strands create complex radial and axial temperature fields. This gap in existing methodologies motivates the use of a three-dimensional finite element analysis (FEA) approach in this study, which can explicitly resolve these internal thermal gradients and provide a more accurate assessment of conductor performance, particularly for advanced energy-saving designs operating in the challenging thermal environments of alpine regions.

As a critical component of overhead transmission lines, the mechanical performance of aluminum core steel-reinforced (ACSR) conductors directly affects the safety and stability of the lines. The internal strand structure of the conductor is complex, with uneven stress distribution among strands under static pre-tension. Operational vibrations and bending further induce dynamic changes in inter-strand forces, and this contact stress is a major cause of conductor wear. Traditional calculation and experimental methods struggle to accurately characterize such internal mechanical behaviors, making finite element analysis an effective approach to studying contact loads between strands. For example, Frigerio et al. [10] used a three-dimensional finite element model to reveal that manufacturing residual stress can cause the inelastic elongation of conductors under low load levels. Notably, the mechanical states of conductors are closely related to their thermal behavior: stress distribution affects heat dissipation and thermal expansion, while temperature changes, in turn, influence the mechanical properties of materials, leading to stress redistribution [11,12,13,14]. Li et al. [15,16] further confirmed this coupling relationship through a three-dimensional finite element model analyzing the temperature and stress distribution under current carrying conditions. Therefore, when studying the characteristics of ACSR conductors, it is essential to comprehensively consider the coupling relationship between stress and temperature, laying the foundation for the subsequent analysis of conductors under electrothermal–mechanical multi-physical-field interactions.

In summary, there is still a lack of systematic research on the operational characteristics of energy-efficient conductors under stress–temperature coupling in cold regions. This study selects three types of energy-efficient conductors and one conventional ACSR conductor, establishing a three-dimensional finite element model based on the actual line conditions in the cold region of Heilongjiang Province, China. Multiple composite working conditions are designed to systematically analyze the operational performance of these conductors under the coupled effects of the environmental temperature and mechanical loads. The research employs finite element methods to simulate the stress and temperature distribution of conductors under actual working conditions, focusing on revealing the influences of the current carrying capacity, ambient temperature, and wind speed on the temperature fields and layered stress distribution of conductors. The aim is to provide a theoretical basis for the dynamic capacity rating, safe operation, and engineering application of energy-efficient conductors in cold regions.

The research deployment of energy-saving conductors in high-altitude and cold regions has important strategic significance but faces scientific challenges. Low-temperature environments can fully leverage their low resistance advantages to improve transmission performance. These regions are rich in clean energy, and high-performance conductors are the core guarantee for building transmission channels and achieving the “dual carbon” goal. However, the thermal–mechanical coupling in extreme environments tests the performance of conductors, and existing standards are difficult to adapt to new conductors, which can lead to misjudgments. Therefore, conducting multi-physics coupling simulations and performance evaluations in high-altitude environments has both engineering relevance and research value.

It should be noted that this study focuses on technical performance evaluation. A full economic assessment via life cycle costing will be addressed in future work.

## 2. Materials and Methods

### 2.1. Conductor Specifications and Material Properties

This study quantitatively defines energy-saving conductors as a new type of conductor that reduces AC resistance by ≥24% compared to traditional steel-cored aluminum-stranded wires (represented by JL/G1A-240/30) at a reference operating temperature of 75 °C under the same outer diameter and rated current carrying capacity. This standard is based on reducing Joule heat losses. After calculation, the AC resistance of the selected JLHA3-275 conductor is reduced by about 26% compared to conventional conductors, which meets the definition of energy-saving conductors.

To ensure the fidelity of thermal modeling and enable meaningful comparisons of different types of overhead line (OHL) conductors, it is necessary to fully characterize the geometry and material composition of each conductor studied. This study mainly focuses on four representative conductor configurations commonly used in modern transmission systems: a high-strength aluminum alloy conductor (JLHA3-275); two variants of aluminum core steel-reinforced (ACSR) conductors, namely JL1/G1A-240/30 and JL/G1A-240/30; and a hybrid steel core–aluminum alloy conductor. Table 1 summarizes the detailed parameters of all four types of wires, prepared based on manufacturer data sheets, Chinese national standards (such as GB/T 1179-2017 [17]), and established industry practices, including the layer configuration, number of stranded wires, core composition, and total cross-sectional area. The specific structural and geometric parameters of the conductors under investigation are summarized in Table 1, where the data for the JLHA3-275 conductor are adapted from our prior conference publication [18].

### 2.2. Material Thermophysical and Electrical Properties

The accurate prediction of the conductor temperature under steady-state or transient load conditions largely depends on the precise definition of material-specific thermophysical and electrical properties. These parameters control Joule heating, thermal conduction within the cross-section of the conductor, and radiative/convective heat exchange with the environment. In this study, four main materials were involved: cold-drawn aluminum (for JL1/G1A), electrolytic tough asphalt (ETP) aluminum (for JI/G1A), Al-Mg-Si high-strength aluminum alloy (for JLHA3 and JL/LHA1), and galvanized steel (G1A core).

The values used in numerical simulations, including DC resistivity at 20 °C temperature coefficient of resistance, skin effect coefficient, solar absorption, thermal emissivity, specific heat capacity, density, and thermal conductivity, are developed based on IEEE standard methods and established engineering conditions. If the surface optical properties (solar absorption and emissivity) exhibit variability due to aging or oxidation, we select representative values corresponding to relatively new conductor surfaces, which is consistent with common practice in thermal rating studies. Table 2 summarizes all material constants used in the finite element model.

### 2.3. Theoretical Calculation of the Radial Temperatures of Conductors

During the operation of overhead transmission lines, the temperature of a conductor rises as a result of internal heat generation, primarily from Joule heating due to the electrical current and external heating from solar irradiance. This elevated temperature relative to the ambient environment drives heat loss via convection and thermal radiation. When the rate of heat input balances the rate of heat dissipation, the conductor reaches a steady-state condition known as thermal equilibrium, at which point its temperature stabilizes. This principle forms the basis for estimating the operating temperatures of energy-efficient conductors. Following the methodology prescribed in the IEEE standards, the thermal equilibrium can be expressed as described in the following.

The conductor’s thermal behavior at equilibrium is described by the following equation:

(1) $$q_{c}+\;q_{r}=\;q_{s}+\;I^{2}R_{ac}$$

In the equation, I represents the allowable current carrying capacity, where $q_{c}$ represents the convective heat dissipation power per unit length of conductor, W/m; $q_{r}$ represents the radiative heat dissipation power per unit length of conductor, W/m; $q_{s}$ represents the solar heat absorption power per unit length of conductor, W/m; and $R_{ac}$ represents the AC resistance per unit length of conductor.

The current carrying capacity of the conductor is as follows:

(2) $$I=\sqrt{\frac{\left(q_{c}+\;q_{r}\;-\;q_{s}\right)}{R_{ac}}}$$

External environmental factors play a critical role in determining both the heat gains and heat losses of overhead conductors. Specifically, higher wind velocities enhance the rate of convective heat transfer between the conductor surface and the ambient air, thereby promoting more effective cooling. On the other hand, greater solar irradiance leads to the increased absorption of radiant energy by the conductor, raising its thermal load.

Convective heat loss occurs through two primary mechanisms: natural and forced convection. Natural convection arises spontaneously due to buoyancy-driven air flow induced by temperature gradients between the conductor and the surrounding air, and it typically dominates under calm or low-wind conditions, especially at lower ambient temperatures. In contrast, forced convection is driven by an externally imposed air flow (e.g., wind), which significantly improves the heat removal efficiency and results in lower conductor operating temperatures. It is worth noting that the cooling performance of natural convection is considerably weaker than that of forced convection; in fact, its effect is roughly equivalent to that of forced convection only when wind speeds are very low, generally between 0.0 and 0.5 m/s.

The Reynolds number represents the relationship between convective heat dissipation and the diameter of the conductor, namely

(3) $$R_{e}=\frac{D\rho_{f}v}{\mu_{f}}$$

(4) $$\mu_{f}=\frac{1.458\times 10^{-6}{\left(T_{\mathrm{film}\;}+273.15\right)}^{1.5}}{T_{\mathrm{film}\;}+383.4}$$

(5) $$\rho_{f}=\frac{1.293-1.525\times 10^{-4}\;h+6.379\times 10^{-9}{\;h}^{2}}{1+0.00367T_{\mathrm{film}\;}}$$

where h represents the altitude of the conductor; $\mu_{f}$ represents the dynamic viscosity of air, N∙s/m^2^; and $\rho_{f}$ represents the air density, $kg/m^{3}$. The Reynolds number is directly proportional to the outer diameter of the conductor. Forced convective cooling is expressed as

(6) $$q_{c}=0.57\pi \lambda_{f}\left(T_{s}-T_{a}\right)Re^{0.485}$$

Under natural convection conditions, with a wind speed of 0 m/s, the expression for the convective heat dissipation power is

(7) $$q_{cm}=3.645\rho_{f}^{0.5}D^{0.75}{\left(T_{s}-T_{a}\right)}^{1.25}$$

Radiative cooling occurs when there is a temperature difference between the conductor and its environment, and heat is exchanged through radiation. The magnitude of the radiative capability affects the radial temperature difference of the conductor. It is calculated using the Stefan–Boltzmann law, with the expression given as:

(8) $$q_{r}=17.8D\varepsilon \left[{\left(\frac{T_{s}+273.15}{100}\right)}^{4}-{\left(\frac{T_{a}+273.15}{100}\right)}^{4}\right]$$

The calculation of the DC resistance is as follows [12]:

(9) $${\;R}_{dc}=R_{20}\left[1+\alpha_{20}\left(T_{s}-20\right)\right]$$

where $R_{dc}$ represents the DC resistance (measured in Ω); $R_{20}$ represents the DC resistance of the conductor at the standard reference temperature of 20 °C (measured in Ω); $\alpha_{20}$ represents the temperature coefficient of resistance at 20 °C (measured in 1/°C and *T_s_* represents the temperature of the conductor (measured in °C).

### 2.4. Multi-Conductor Electrothermal Modeling Strategy

To accurately capture the non-uniform temperature distribution within the complex stranded geometry of each conductor type, a strand-by-strand electrothermal modeling strategy was adopted. The key challenge lies in the differing electrical and thermal properties of the constituent materials across the four conductor types. For instance, the JLHA3-275 conductor is composed entirely of Al-Mg-Si alloy strands, whereas the JL/G1A and JL1/G1A types feature a central galvanized steel core surrounded by EC-grade or hard-drawn aluminum strands, respectively. The JL/LHA1 conductor presents a hybrid structure with an aluminum alloy outer layer over a steel core.

For each conductor model, the total load current was distributed among its individual strands based on their relative electrical conductance, following the principle that current prefers paths of power resistance. The volumetric heat generation rate (Q) for each strand was then calculated from its assigned current and material resistivity. Critically, for the all-aluminum JLHA3 conductor, this results in a more uniform internal heat generation profile compared to the steel-cored variants, where the high-resistivity steel core contributes negligibly to heat generation but significantly impacts the overall thermal conductivity. These calculated heat generation rates were applied as internal heat sources within their respective strand volumes in the finite element model, ensuring a physics-based representation of Joule heating for each unique conductor architecture.

### 2.5. Calculation of Layered Stress in the Conductor

The combined effects of tension and temperature induce axial elongation in overhead conductors. To facilitate consistent conductor deformation analysis, three key assumptions are formulated:

1. During the deformation process, the conductor satisfies the plane assumption, meaning that the axis of each strand lies within the same circular cross-section and that strands within the same layer are subjected to equal forces.
2. The aluminum core steel-reinforced (ACSR) conductor remains within the elastic phase throughout the deformation process.
3. The effects of compression and friction between strands are neglected.

Under the influence of temperature and tension, energy-saving conductors satisfy deformation coordination in the axial direction, meaning that all strands have the same axial elongation rate:

(10) $$\varepsilon_{n}=\varepsilon =\frac{\partial u}{\partial x}=\frac{T}{EA}+\alpha \left(t_{i}-t_{0}\right)$$

where T represents the tension of the conductor; E denotes the composite elastic modulus of the conductor; A is the cross-sectional area of the conductor; α represents the axial composite thermal expansion coefficient of the conductor; $t_{i}$ is the temperature of the conductor in a specific state; and $t_{0}$ represents the manufacturing temperature of the conductor, which is generally set at 20 °C.

The temperature only causes the elongation of the conductor strands, without generating thermal stress. Therefore, under the action of tension, the strain of each layer of strands in the conductor is:

(11) $$\varepsilon_{n}^{*}=\left({cos}^{2}\;\beta_{n}-\mu_{n}{sin}^{2}\;\beta_{n}\right)\varepsilon -\alpha_{i}\left(t_{i}-t_{0}\right)$$

where $\mu_{n}$ represents the Poisson’s ratio of the strands in the *n*-th layer, and $\beta_{n}$ refers to the lay angle (twist angle), which is the angle formed between the strands winding around the inner core conductor axis and the axial direction.

The axial stress of the strands in the *n*-th layer is

(12) $$\sigma_{n}=E_{n}\varepsilon_{n}^{*}$$

The total axial tension of the conductor is:

(13) $$T=\sum_{n=1}^{n}\;A_{n}\sigma_{n}=\sum_{n=1}^{n}\;A_{n}E_{n}\varepsilon_{n}^{*}cos\;\beta_{n}$$

where $A_{n}$ is the total cross-sectional area of the strands in the *n*-th layer.

Given that $t_{1}$ is the average stress of a conductor at a lower temperature $\sigma_{01}$, according to the state equation of overhead lines with unequal suspension heights (14), $t_{2}$ is the average stress of the conductor at a temperature $\sigma_{02}$ [16]:

(14) $$\sigma_{02}-\frac{E\gamma_{2}^{2}l^{2}{cos}^{3}\;\beta }{24\sigma_{02}^{2}}=\sigma_{01}-\frac{E\gamma_{1}^{2}l^{2}{cos}^{3}\;\beta }{24\sigma_{01}^{2}}-\alpha Ecos\;\beta \left(t_{2}-t_{1}\right)$$

where $\sigma_{01},\sigma_{02}$ represent the stress at the lowest point of the sag in the overhead lines under two different conditions; $\gamma_{1},\gamma_{2}$ represent the specific loading of the overhead lines under two different conditions; $t_{1},t_{2}$ represent the temperatures of the overhead lines under two different conditions; l,β represent the span length and height difference angle of the section, respectively; and α,E represent the overall axial thermal expansion coefficient and the comprehensive elastic modulus of the overhead line, respectively.

At *t*_2_, the total tension of the conductor is

(15) $$T=\sigma_{02}\sum_{n=1}^{n}\;A_{n}$$

By incorporating actual engineering data into the conductor state Equation (14), the conductor tension at a specified temperature is determined, as expressed in Equation (15). This calculated tension is then utilized to compute the axial strain of the conductor using Equation (10). The resulting axial strain is subsequently substituted into Equation (11) to calculate the strand-wise strain for each layer of strands. Finally, these strand-wise strains are incorporated into Equations (12) and (13) to derive the axial stress and tension of each layer of strands within the conductor.

### 2.6. Unified Finite Element Framework for Comparative Analysis

**Figure 1:**
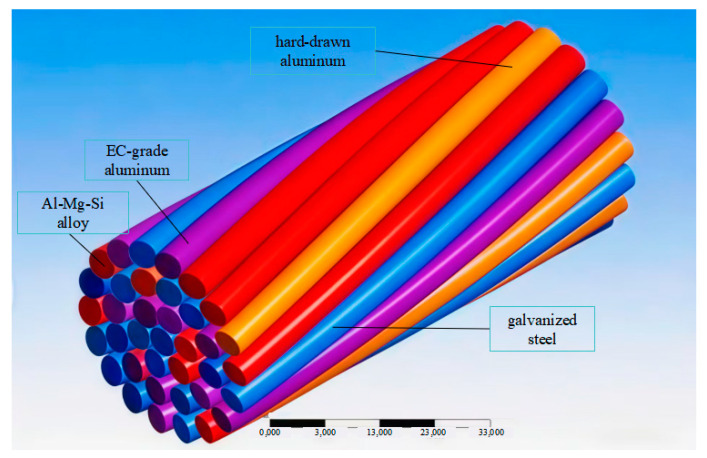
Model of the aluminum alloy stranded conductor. Adapted from Ref..

A unified three-dimensional finite element framework was established to enable a direct and consistent comparison of the thermal and mechanical performance of the four selected conductors under identical environmental loads. The primary objective was to faithfully replicate the distinct helical lay patterns and inter-strand air gaps characteristic of each conductor type, as these microstructural features critically influence both heat transfer pathways and stress distribution. The finite element model is shown in Figure 1.

The geometric models were constructed to match the exact specifications provided in Table 3, including the number of layers, strand count per layer, strand diameter, and lay ratio. Special attention was paid to the core region: a solid cylindrical domain was used for the monolithic JLHA3 conductor, while a seven-wire stranded bundle was modeled for the steel cores of the other three conductor types. The interstitial air gaps—naturally formed during the helical stranding process—were explicitly retained in all models, as they act as thermal insulators and significantly affect inter-strand contact mechanics.

Each conductor consists of multiple concentric layers of aluminum alloy or pure aluminum strands wound helically around a central core, which is either solid (in the case of JLHA3) or stranded steel (for the ACSR-type conductors). The specific materials include Al-Mg-Si alloy, EC-grade aluminum, hard-drawn aluminum, and galvanized steel, with properties assigned according to experimental data.

It should be noted that the colors in Figure 1 are automatically generated by the finite element software solely for visual distinction of different material components and structural layers; they do not represent actual physical colors of the conductors. The resulting models were then subjected to coupled electrothermal–structural analysis: the electrical problem was first solved to obtain the steady-state temperature field, which was subsequently applied as a thermal load in the structural analysis to evaluate the stress distribution within each conductor.

### 2.7. Validation of the Finite Element Model of Energy-Efficient Conductors

**Figure 2:**
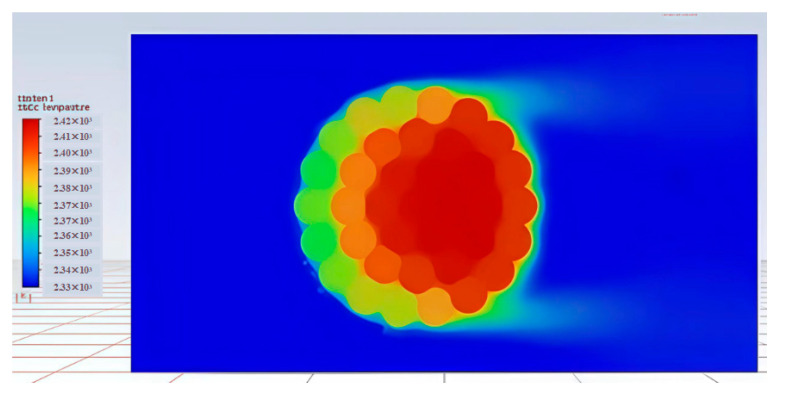
Radial temperature contour map of the JLHA3-275 conductor at a current load of 400 A under an ambient temperature of −40 °C. Adapted from Ref..

**Figure 3:**
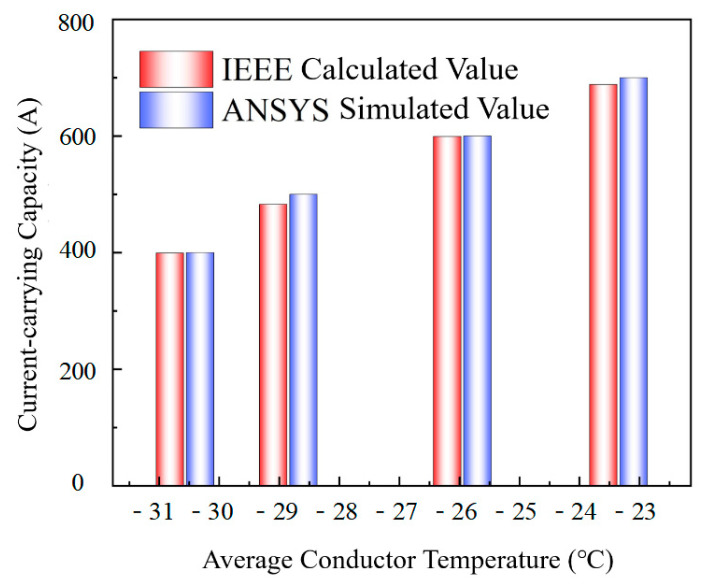
Comparison between simulation values and IEEE calculation values.

The finite element model was validated using the JLHA3-275-37 conductor as a test case, under conditions of a −40 °C ambient temperature, a 1 m/s wind speed, and a 500 A current carrying capacity. This validation procedure, including the specific environmental conditions and the comparison with the IEEE standard method, follows the approach established in our previous conference publication. The simulated radial temperature distribution is shown in Figure 2, and a detailed comparison between the simulation results and the IEEE-calculated values is presented in Figure 3. The close agreement between the two methods confirms the finite element model’s accuracy in replicating the thermal behavior of the conductor, thereby providing a reliable foundation for the comparative performance evaluation conducted in this study. Overall, the central area presents a bright red to yellow color, indicating that this is the peak temperature of the high-temperature zone. Moving radially outward from the center, the color gradually transitions from green and cyan to dark blue, reflecting a gradual decrease in temperature to the ambient level. The concentric and slightly petal shaped thermal gradient indicates that heat is conducted from the local heat source to the surrounding medium, and the asymmetry of the temperature distribution may be influenced by material properties, boundary conditions, or external cooling mechanisms.

As shown in Table 4, the calculated results of the finite element model developed in this study do not exceed 3.88% of the IEEE theoretical values. This error falls within the engineering tolerance range of 5%, indicating that the model results have a high degree of reliability. Therefore, the finite element simulation method provides a more precise analysis of a conductor’s radial temperature distribution. Compared to the IEEE standard method, it more accurately represents real-world operating conditions. This is because it fully accounts for heat sources, dissipation mechanisms, and transfer pathways, leading to more precise results. Conversely, the IEEE method, which necessitates extensive calculations and mainly estimates the permissible current based on known conductor temperatures, is limited in effectively evaluating the true temperature distribution within the conductor.

## 3. Influences of Key Factors on Radial Temperature of Energy-Saving Conductor in Alpine Region

### 3.1. Influence of Ampacity on Energy-Saving Conductor

This study selected extremely conservative benchmark environmental conditions to evaluate the applicability of energy-saving conductors in high-altitude regions: an extremely low temperature of −40 °C was used to verify the mechanical strength, and a low wind speed of 1 m/s (weakening convective heat dissipation and raising the conductor temperature rise) and maximum solar irradiance of 1000 W/m^2^ formed the most unfavorable thermal performance scenario. Although this combination is not a daily operating condition, it meets the limit performance verification criteria for transmission equipment and can effectively reveal the performance differences of conductors under severe conditions.

**Figure 4:**
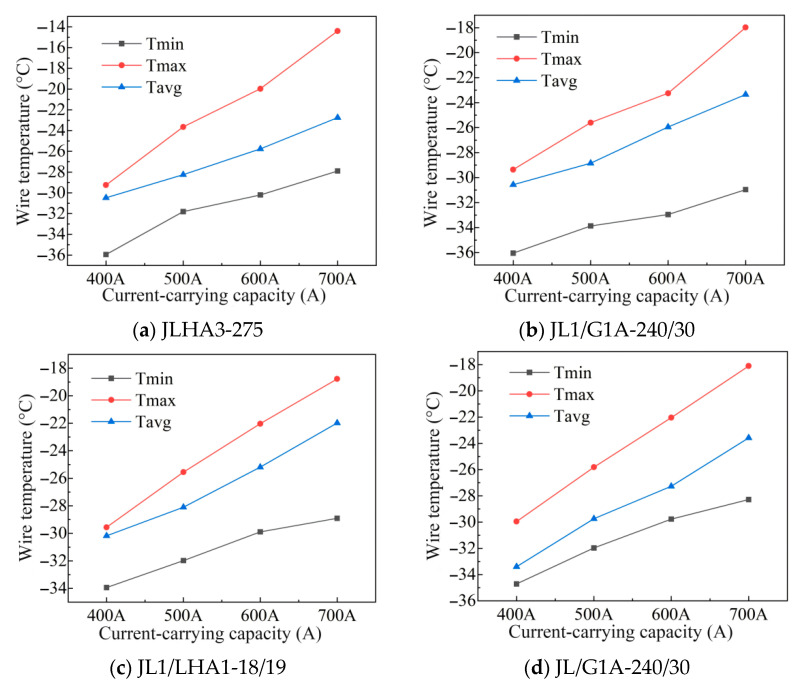
Radial temperature diagrams of four types of conductors under different current carrying capacities.

Through the verification and analysis of the above finite element model, the effectiveness and feasibility of the model have been proven. The following is the analysis and comparison of the main influencing factors of the medium-strength aluminum alloy conductor, including the radial temperature field simulation analysis of the carrying capacity, wind speed, and ambient temperature. When the wind speed is 1 m/s and the light intensity is 1000 w/m^2^, the conductor temperature changes at 400 A, 500 A, 600 A, and 700 A (100 A as steps) are calculated, as shown in Figure 4.

At 400 A ampacity, the radial temperatures of the four types of conductors are lower than −30 °C. Among them, the temperature of the all-aluminum-alloy conductor JLHA3-275 is the lowest, which is −30.57 °C—i.e., 0.39 °C lower than that of the steel-cored aluminum strand conductor JL/G1A, indicating that it has better heat dissipation performance under low load conditions. With the carrying capacity gradually increased from 400 A to 700 A, the temperature rise of JLHA3-275 was 7.22 °C, which was significantly lower than that of JL/G1A at 8.43 °C. This difference reflects that the medium-strength aluminum alloy conductor has better thermal stability under high current carrying conditions, and its smaller temperature rise is conducive to maintaining the structural integrity and electrical performance of the conductor in long-term high-load operation. This phenomenon may be due to the high thermal conductivity of aluminum alloy and the uniformity of its cross-section structure, which allows the heat to be more effectively diffused outward, thus inhibiting the sharp rise in the temperature, being especially suitable for transmission scenarios in high and cold regions sensitive to temperature rises.

### 3.2. Impact of Wind Speed on Energy-Saving Conductor

**Figure 5:**
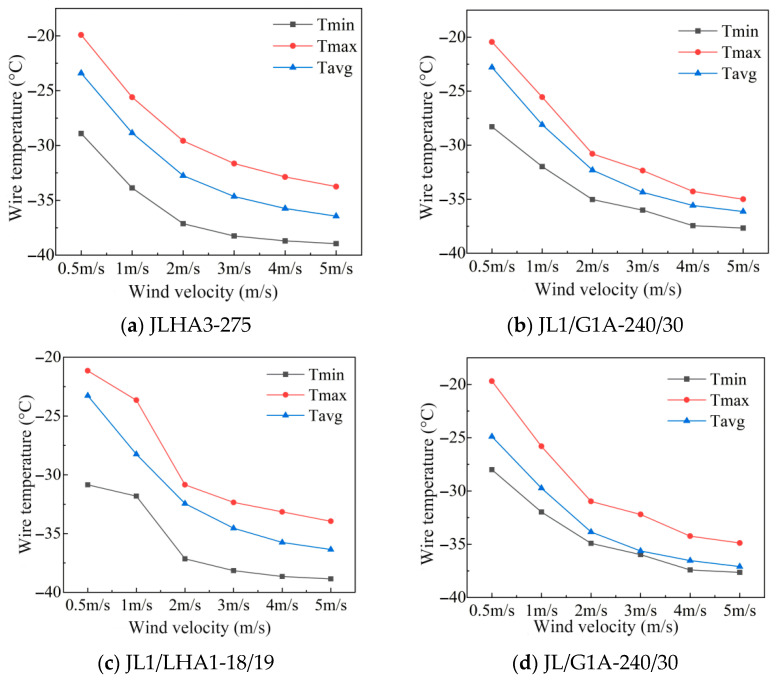
Radial temperature diagrams of four types of conductors under different wind speeds.

When the current carrying capacity is 500 A, the ambient temperature is −40 °C, and the solar radiation is 1000 w/m^2^, the temperature changes of the conductor with wind speeds of 0.5 m/s, 1 m/s, 2 m/s, 3 m/s, 4 m/s, and 5 m/s (1 m/s as steps) are calculated, as shown in Figure 5.

The JL1/LHA1 and JLHA3-275 conductors show more significant temperature difference convergence trends under high wind speeds. When the wind speed is low (≤2 m/s), the temperature of LJHA3-275 is −32.75 °C, which is 0.85 °C lower than that of JL/G1A. This temperature difference shows that it can still maintain a good heat dissipation capacity under weak wind conditions, which is helpful to improve the actual current carrying potential of the line. When the wind speed is high (such as 5 m/s), the temperature of JL1/LHA1 is −36.35 °C, which is only 0.1 °C lower than the temperature of −36.45 °C for JLHA3-275, indicating that the two temperatures tend to be close under a strong wind cooling effect. However, in actual project selection, it is still necessary to perform comprehensive evaluation and selection based on the frequency distributions of different wind speed ranges, the line operation economy, long-term reliability, and other factors.

### 3.3. Impact of Ambient Temperature on Energy-Saving Conductor

**Figure 6:**
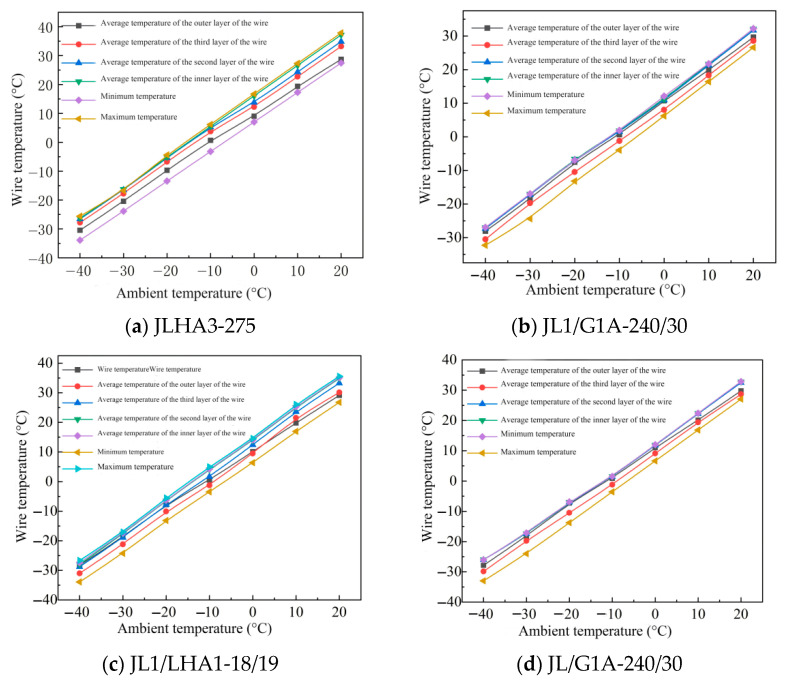
Radial temperature diagrams of four types of wires under different ambient temperatures: (**a**) JLHA3-275; (**b**) JL1/G1A-240/30; (**c**) JL/LHA1-135/140; (**d**) JL/G1A-240/30. (**a**) is adapted from Ref..

When the current carrying capacity is 500 A, the wind speed is 1 m/s, and the solar radiation is 1000 w/m^2^, the temperature changes of the conductor under wind speeds of −40, −30, −20, −10, 0, 10, and 20 °C (10 °C as steps) are calculated, as shown in Figure 6.

The temperature difference between the inner and outer layers of steel-cored aluminum strand conductors JL/G1A and JL1/G1A is relatively consistent under all test ambient temperatures, indicating that their structures have good temperature adaptability and stable temperature resistance characteristics, which are conducive to maintaining the stability of electrical performance in an environment with significant temperature differences. Under the extremely low temperature of −40 °C, the temperature rise of the all-aluminum-alloy conductor JLHA3-275 is 11.15 °C, which is lower than the temperature of 12.15 °C for JL/G1a, where the temperature difference is 1.0 °C. This result further confirms that JLHA3-275 has better heat dissipation performance and lower resistance effects in extremely low-temperature environments, making it especially suitable for application scenarios with strict requirements for conductor heat dissipation in alpine regions, and this can improve the energy efficiency of the system while ensuring the safety of transmission.

### 3.4. Quantification of Energy-Saving Benefits Based on Temperature Reduction During Operation

To convert the simulated temperature differences of conductors into actual energy-saving benefits, this study quantitatively evaluated the transmission loss based on Joule’s law. Under the same current carrying condition of 700 A, the AC power loss, *P_loss_*, of a conductor can be expressed as:

(16) $$P_{loss}=\;I^{2}R_{ac}\left(T\right)$$

Here, *I* = 700 A is the working current, and *R_ac_* (*T*) is the AC resistance, which depends on the average operating temperature T. According to the simulation results in Section 3.1, the average operating temperature of JLHA3-275 is −23.35 °C, while that of the conventional JL/G1A-240/30 conductor is −21.75 °C. Due to the linear variation in resistivity with temperature, lower operating temperatures directly result in a lower *R_ac_*. The power loss data of different conductors with a current carrying capacity of 700 A are listed in Table 5 below.

By calculation, it can be concluded that, under the above operating conditions, the unit length power loss of JLHA3-275 is reduced by about 16.7% compared to conventional conductors. To evaluate its annual benefits, assuming that a 100 km transmission line operates at this load level (or an equivalent average load) throughout the year, the JLHA3-275 conductor can save over 800 MWh of electricity annually. This quantitative result clearly indicates that a slight decrease in operating temperature (about 1.6 °C) can be converted into significant energy savings and carbon reduction benefits by reducing resistance losses, fully verifying the enormous economic and environmental value of using energy-saving conductors in high-altitude regions.

This quantitative comparison clearly indicates that the JLHA3-275 energy-saving conductor not only has energy-saving advantages due to its lower intrinsic resistivity under actual operating conditions in high-altitude regions, but also further benefits from the decrease in resistance caused by its lower operating temperature, thereby achieving a significant reduction in line losses. A 23.9% reduction in power loss means that, under the same transmission task, the power grid will consume less electricity to overcome line impedance, directly improving the energy utilization efficiency.

## 4. Stress Analysis of Energy-Saving Conductor in Alpine Region

### 4.1. Mechanical Stress Response Under Thermal Loading

**Figure 7:**
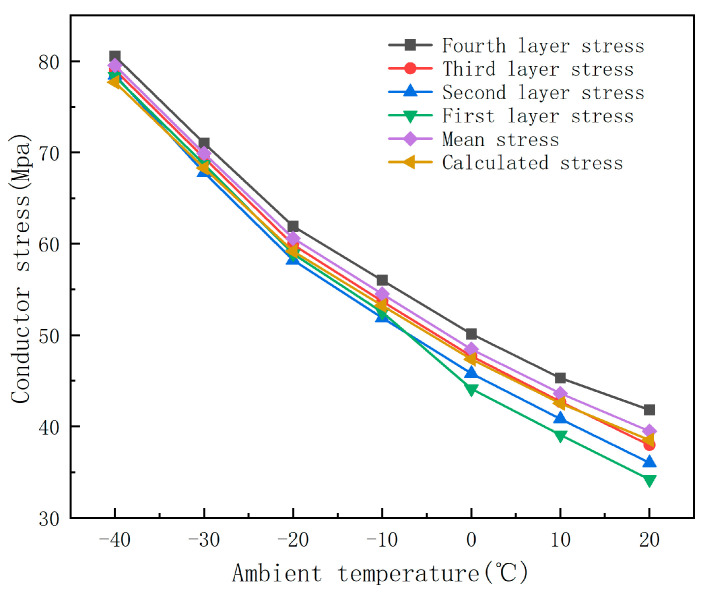
Stress distribution across different layers of a medium-strength aluminum alloy conductor.

**Figure 8:**
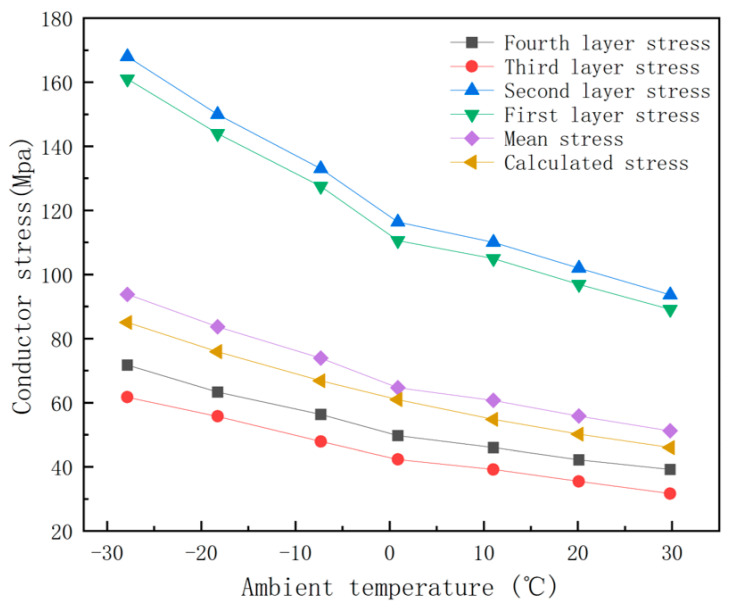
Stress distribution across different layers of a steel core aluminum-stranded conductor.

In this study, under the conditions of a wind speed of 1 m/s, a current carrying capacity of 500 A, and an average conductor temperature in the range of −40 °C to −20 °C, the stress distribution in different layers and sections of the conductor is systematically analyzed. Figure 7 and Figure 8 show that the stresses of each layer in the JLHA3 and JL1/LHA1 conductors show a uniform downward trend with an increase in temperature. In particular, in the outermost layer, the stress decreases from 80.53 MPa to 41.81 MPa, which shows that the conductor has high mechanical stability in low-temperature environments. In contrast, the stress of the steel core in the JL/G1A and JL1/G1A conductors is significantly higher than that of the aluminum strand layer, indicating that the structural stability of the steel core is more prominent at low temperatures. Specifically, the stresses of the second layer and the inner layer of the steel core decreased from 168 MPa to 93.6 MPa, indicating that this type of conductor has better resistance to physical deformation due to its enhanced stress tolerance at extremely low temperatures.

**Figure 9:**
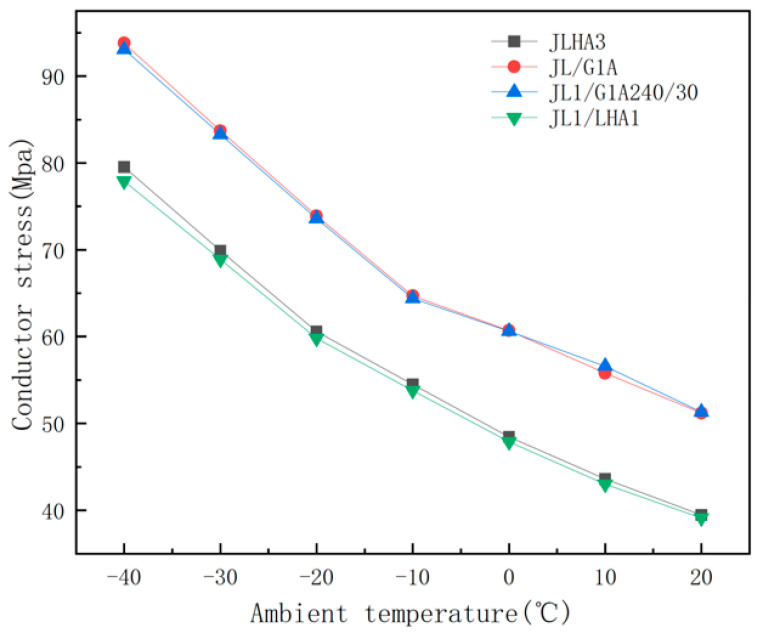
Comparative diagram of stresses in four types of conductors under different ambient temperatures.

Figure 9 shows the results of the finite element stress analysis: with a change in ambient temperature, JL/G1A and JL1/G1A240/30 show high stress values at all temperature monitoring points. At −40 °C, the stress reaches 93.8 MPa and 93.1 MPa, respectively, which indicates that the conductor has strong structural stability at extremely low temperatures but also that it may have potential vulnerabilities caused by thermal stress. In contrast, the stress levels of JLHA3 and JL1/LHA1 in the same temperature range were lower: they decreased from 79.53 MPa and 77.9 MPa to 39.46 MPa and 39.1 MPa, respectively. This low stress response effectively reduces the risk of damage caused by temperature fluctuations, thereby improving the adaptability and durability of these conductors in cold environments.

The above analysis further reveals that there are significant differences in stress distribution between conductors of different structural types at low temperatures, which is mainly due to the interaction between material properties and composite structures. For aluminum alloy conductors (such as JLHA3 and JL1/LHA1), the stress response shows a uniform and gentle downward trend with an increase in temperature, which is mainly due to the relatively consistent thermodynamic properties of aluminum alloy materials and the coordination of stress transfer between layers. This gentle stress temperature relationship is conducive to maintaining the overall stability of the conductor structure in an environment with frequent temperature fluctuations, reducing the cumulative damage caused by the thermal cycle. This is more suitable for long-term operation in areas where the temperature difference changes significantly but the temperature is still considered low.

Comparatively speaking, ACSR conductors (such as JL/G1A and JL1/G1A) show stronger deformation resistance under extremely low temperature conditions, which is mainly due to the high strength and high modulus of elasticity of the steel core. The steel core can still maintain a high stress bearing level in the range of 40 °C to 20 °C, which effectively inhibits the overall deformation of the conductor and improves the mechanical reliability of the line in severe cold environments. However, the difference in the thermal expansion coefficient between the steel core and aluminum strand layer may cause the concentration of interfacial stress, especially when the temperature changes sharply. This local stress peak can easily lead to the accumulation of micro-fatigue damage, which may affect the fatigue life and fracture toughness of the conductor under long-term action.

Therefore, in the selection and safety assessment of transmission lines in low-temperature areas, it is necessary to establish a comprehensive trade-off based on the specific environmental conditions and operation requirements. If continuous low temperatures and extreme climates are taken as the main consideration, the ACSR conductor has more advantages, with its excellent deformation resistance. If the ambient temperature fluctuates frequently and the temperature difference is large, the aluminum alloy conductor may be more suitable, with its good stress coordination and temperature fluctuation resistance. Through quantitative stress analysis, this study provides an important theoretical basis and data support for the material selection, structural design, and safe operation and maintenance of conductors under different temperature conditions, and it will help to realize the economic, safe, and stable operation of transmission lines in cold environments.

### 4.2. Comprehensive Performance Comparison

To comprehensively evaluate the performance of the four typical overhead conductors under operating conditions in high-altitude regions, this study conducted a systematic analysis from two dimensions: thermodynamics and mechanics. Section 3 reveals the radial temperature distribution characteristics of the different conductors under the same current carrying conditions through a three-dimensional finite element model, indicating that JLHA3-275 has a lower steady-state operating temperature and better thermal stability. Section 4 further investigates the effects of sudden changes in ambient temperature on the axial stress of the conductor, and the results show that the all-aluminum-alloy conductor JLHA3-275 exhibits significantly lower and more evenly distributed thermally induced stress, demonstrating better mechanical adaptability. In order to facilitate an intuitive and quantitative comparison of the comprehensive performance of various conductors in terms of the current carrying capacity, thermal performance, mechanical response, and energy efficiency, key performance indicators, including the average operating temperature, maximum axial stress, relative AC resistance, transmission loss, and current carrying capacity, are summarized in Table 6, considered under uniform boundary conditions (700 A current, −40 °C ambient temperature, 1 m/s wind speed, 1000 W/m^2^ solar radiation), in order to provide a clear decision-making basis for conductor selection in high-altitude cold regions.

As summarized in Table 6, the JLHA3-275 conductor demonstrates superior integrated performance, exhibiting the lowest operating temperature, minimal transmission loss, and the most favorable mechanical stress characteristics among all evaluated conductors.

## 5. Conclusions

The grid-level benefits of energy-saving conductors are profound: wide application in high-altitude corridors can save hundreds of GWh annually and boost system energy efficiency; lower operating temperatures enhance the line implicit capacity to enhance transmission without extra towers, facilitating western clean energy export; and reduced line losses improve voltage quality and ensure the stable operation of remote grids. Conductor cooling reflects material advantages and acts as a key pivot for grid energy efficiency upgrading and energy transformation.

An analysis of the temperature fields for four conductor types shows that JLHA3 has optimal heat dissipation and low resistance loss characteristics across various load conditions in cold regions. At 700 A, JLHA3’s radial temperature drops to −23.35 °C, i.e., 1.6 °C lower than those of conventional conductors, demonstrating improved heat dissipation and resistance loss control. Increased wind speeds yield a substantial decrease in conductor temperature; notably, at 5 m/s, that of JLHA3 reaches −36.45 °C, outperforming JL/G1A at −35.98 °C, thereby demonstrating enhanced heat dissipation capabilities. This temperature decrease is accompanied by reduced resistance losses, leading to significant improvements in power transmission efficiency. Moreover, JLHA3 exhibits high thermal stability across a broad temperature range of −28.85 °C to 29.03 °C, ensuring stable electrical performance in cold regions with large temperature fluctuations. Its exceptional thermal stability, heat dissipation, and low resistance loss characteristics render JLHA3 an ideal choice for energy-efficient power transmission in cold climates, effectively minimizing energy losses and enhancing overall system reliability and efficiency.

An analysis of the stress distribution across the four conductor types reveals that JLHA3 and JL1/LHA1 display uniform stress variation with rising temperatures, characterized by decreased stress levels from 79.53 MPa and 77.9 MPa to 39.46 MPa and 39.1 MPa, respectively. This temperature-dependent stress reduction underscores their flexibility and adaptability. In contrast, JL/G1A and JL1/G1A exhibit elevated stress values, peaking at 93.8 MPa and 93.1 MPa at −40 °C, indicating strong structural stability but also increased thermal stress, potentially requiring reinforced support structures in extremely cold conditions. Notably, JLHA3 demonstrates superior thermal adaptability and flexibility due to its uniform stress distribution and lower overall stress levels under temperature fluctuations, making it a prime candidate for power transmission applications in cold regions.

This study has confirmed the performance advantages of the JLHA3 conductor across a wide range of environmental conditions through parametric analysis. Future work will incorporate long-term, site-specific meteorological time series to perform a probabilistic assessment for the more precise quantification of its annual energy savings and dynamic capacity benefits.

## Figures and Tables

**Table 1:** Conductor model parameters.

Conductor Model	Type	Material Composition	Steel Core	Aluminum/ Alloy Layers	Total Cross-Sectional Area (mm^2^)	Outer Diameter (mm)
JLHA3-275	Medium-strength aluminum alloy stranded wire	AI-Mg-Si Alloy	-	4 Layers: Inner layer: 1 piece, diameter 3.08 mm Second layer: 6 pieces, lay ratio 16 Third layer: 12 pieces, lay ratio 14 Fourth layer: 18 pieces, lay ratio 12	~275	~21.6
JL1/G1A-240/30	Steel-cored hard aluminum stranded wire	Hard-Drawn Aluminum	Layer 1: −7 steel wires (typical structure)	2 Layers: Inner layer: 24 aluminum wires Outer layer: 26 aluminum wires	240 (AL) 30 (Steel)	~21.6
JL/LHA1-135/140	Steel-cored aluminum alloy stranded wire	Aluminum Alloy	Layer 1: −7 steel wires (typical structure)	2 Layers: Inner layer: 16–18 aluminum alloy wires Outer layer: 24–26 aluminum alloy wires	135 (Alloy) 140 (Steel)	~22.0
JL/G1A-240/30	Steel-cored aluminum stranded wire (conventional conductor)	EC Grade Aluminum	Layer 1: −7 steel wires (typical structure)	2 Layers: Inner layer: 24 aluminum wires Outer layer: 26 aluminum wires	240 (AL) 30 (Steel)	~21.6

**Table 2:** Detailed material parameters.

Material Type	Hard-Drawn Al, JL1	EC-Grade Al, JL	AI-Mg-Si Alloy, JLHA3/LHA1	Galvanized Steel, G1A
Direct current at 20 °C Resistance rate ρ_20_ (Ω·m)	2.8264 × 10^−8^	2.8264 × 10^−8^	3.2500 × 10^−8^	1.3800 × 10^−7^
Temperature coefficient of resistance α (1/°C)	0.00403	0.00403	0.00360	0.00450
Epidermal effect coefficient	1.02 (at 700 A)	1.02 (at 700 A)	1.01 (at 700 A)	-
Solar energy absorption rate (*ɛ_s_*)	0.50–0.60	0.20–0.30	0.30–0.40	0.60–0.80
Thermal emissivity (*ɛ_r_*)	0.20–0.30	0.05–0.10	0.10–0.20	0.60–0.80
Specific heat capacity *c_p_* (J/(kg·K))	900	900	880	480
Density ρ (kg/m^3^)	2700	2700	2700	7800
Thermal conductivity k (W/(m·k))	205	230	180	45

**Table 3:** Structure of the medium-strength aluminum alloy conductor.

Wire Material	Strand Configuration (Layer/Root)	Wire Diameter (mm)	Lay Ratio	Pitch Length (mm)
Aluminum alloy	1 layer, 1 root	3.08	-	-
	2 layers, 6 roots		16	147.06
	3 layers, 12 roots		14	217.39
	4 layers, 18 roots		12	259.07

**Table 4:** Error comparison between IEEE theoretical values and simulation values.

Average Conductor Temperature (°C)	IEEE-Calculated Value (°C)	FE Simulation (°C)	Relative Error (%)
−30.57	399.41	400.00	−0.15
−28.85	482.80	500.00	−3.44
−25.95	599.26	600.00	−0.12
−23.35	688.48	700.00	−1.65

**Table 5:** Comparison of power losses of different conductors under 700 A current carrying capacity.

Conductor Type	Average Operating Temperature (°C)	Power Loss Per Unit Length (M/m)	Loss Reduction Relative to JL/G1A (%)
JLHA3-275	−23.35	36.64	23.9
JL/G1A-240/30	21.75	48.13	0.0

**Table 6:** Comparison of key performance indicators for four typical conductors under uniform boundary conditions.

Performance Metric	JLHA3-275	JL1/G1A-240/30	L/LHA1-135/140	JL/G1A-240/30
Ampacity	700	700	700	700
Radial average temperature (°C)	−23.35	−21.75	~−22.50	−21.75
Maximum axial stress (MPa)	39.46	93.1	~85.0	93.8
Relative AC resistance (%)	100.0	~112.5	~108.0	~112.5
Relative transmission loss (%)	100.0	~116.7	~110.0	~116.7
Thermal stability	Excellent	Good	Good	Good
Mechanical adaptability	Excellent	Moderate	Good	Moderate

## Data Availability

The original contributions presented in this study are included in the article. Further inquiries can be directed to the corresponding author.
